# A quality improvement project on timely completion of bloods and ECGs on a tier 4 child and adolescent inpatient unit

**DOI:** 10.1192/bjo.2021.579

**Published:** 2021-06-18

**Authors:** Emma Salter, Philippa Snow, Kiera Friel, Nicola Biddiscombe

**Affiliations:** Somerset Partnership NHS Foundation Trust

## Abstract

**Aims:**

Physical health monitoring is paramount to optimal care for psychiatric patients. Blood tests and ECGs are invaluable tests throughout a patient's care. At baseline, they aid investigation of potential organic causes of psychiatric presentations and provide organ and electrolyte status before starting medication. Common psychotropic medications carry physical health risks: bloods and ECGs aid in monitoring potential side effects of prescribed medication.

In this local Tier 4 inpatient unit, anecdotal observation revealed completion of these basic investigations was noted to be suboptimal.

This project aimed to improve timely completion of baseline (within 72 hours of admission) and monitoring (within one week of due date) bloods and ECGS.

**Method:**

This project was completed within a 12-bed child and adolescent inpatient unit. Using Plan Do Study Act (PDSA) methodology, the multidisciplinary team collated driver diagrams to identify potential areas for intervention. Following baseline analysis, colleague communication was considered key. Consequently, a chart for bloods and ECG completion was created.

Each monthly PDSA cycle included the following consecutive interventions:

- PDSA cycle 1: chart implementation
- PDSA cycle 2: chart simplification and font size increase
- PDSA cycle 3: allocated change in team leader for this cycle
- PDSA cycle 4: Blood request pocket in office
- PDSA cycle 5: chart simplification through removal of dates
- PDSA cycle 6: ECG pocket
- PDSA cycle 7: box on handover list

**Result:**

Monthly investigations and admission numbers are unpredictable and inconsistent in this cohort: relevant case numbers per PDSA ranged from zero to ten. The results were presented as percentages to allow for direct comparison between cycles.

Baseline and results of each consecutive PDSA cycle described above were as follows (N/A represents a cycle where no investigations were required):

Admission bloods were completed within 72 hours in 50%, 100%, 50%, 80%, N/A, 100%, 100%, 100%

Admission ECG was completed within 72 hours in 30%, 66%, 50%, 70%, N/A, 100%, 100%, 100%

Monitoring bloods were completed within one week of due date in 25%, 33%, 0%, 80%, 100%, 100%, 100%, 100%

Monitoring ECG was completed within one week of due date in 0%, 0%, N/A, 66%, 100%, 66%, N/A 100%

**Conclusion:**

Through close multidisciplinary collaboration and chart implementation, completion of bloods and ECGs improved. Low patient numbers per PDSA cycle resulted in large changes in percentage results, limiting the significance of these findings. Wider implementation of the chart within local Trust inpatient wards is considered.

